# Differential Noradrenergic Modulation of Monetary Reward and Visual Erotic Stimulus Processing

**DOI:** 10.3389/fpsyt.2018.00346

**Published:** 2018-07-31

**Authors:** Heiko Graf, Maike Wiegers, Coraline D. Metzger, Martin Walter, Birgit Abler

**Affiliations:** ^1^Department of Psychiatry and Psychotherapy III, Ulm University, Ulm, Germany; ^2^Department of Psychiatry, Otto von Guericke University, Magdeburg, Germany; ^3^Institute of Cognitive Neurology and Dementia Research, Otto von Guericke University, Magdeburg, Germany; ^4^German Center for Neurodegenerative Diseases, Bonn, Germany; ^5^Department of Psychiatry, Eberhard Karls University, Tuebingen, Germany

**Keywords:** fMRI, primary reward, secondary reward, reboxetine, amisulpride, healthy, money, erotic

## Abstract

We recently investigated the effects of the noradrenergic antidepressant reboxetine and the antipsychotic amisulpride compared to placebo on neural correlates of primary reinforcers by visual erotic stimulation in healthy subjects. Whereas, amisulpride left subjective sexual functions and corresponding neural activations unimpaired, attenuated neural activations were observed under reboxetine within the nucleus accumbens (Nacc) along with diminished behavioral sexual functioning. However, a global dampening of the reward system under reboxetine seemed not intuitive considering the complementary role of the noradrenergic to the dopamine system in reward-related learning mediated by prediction error processing. We therefore investigated the sample of 17 healthy males in a mean age of 23.8 years again by functional magnetic resonance imaging (fMRI), to explore the noradrenergic effects on neural reward prediction error signaling. Participants took reboxetine (4 mg/d), amisulpride (200 mg/d), and placebo each for 7 days within a randomized, double-blind, within-subject cross-over design. During fMRI, we used an established monetary incentive task to assess neural reward expectation and prediction error signals within the bilateral Nacc using an independent anatomical mask for a region of interest (ROI) analysis. Activations within the same ROI were also assessed for the erotic picture paradigm. We confirmed our previous results from the whole brain analysis for the selected ROI by significant (*p* < 0.05 FWE-corrected) attenuated activations within the Nacc during visual sexual stimulation under reboxetine compared to placebo. However, activations in the Nacc concerning prediction error processing and monetary reward expectation were unimpaired under reboxetine compared to placebo, along with unimpaired reaction times in the reward task. For both tasks, neural activations and behavioral processing were not altered by amisulpride compared to placebo. The observed attenuated neural activations within the Nacc during visual erotic stimulation along with unimpaired neural prediction error and monetary reward expectation processing provide evidence for a differential modulation of the neural reward system by the noradrenergic agent reboxetine depending on the presence of primary reinforcers such as erotic stimuli in contrast to secondary such as monetary rewards.

## Introduction

The prospect of rewards is fundamental for human motivation, goal-directed behavior and learning (1). Classical models differentiate innate primary rewards such as food, sex and shelter, from acquired secondary rewards, i.e., money or power (2). Considering the reinforcement learning model (3), learning about reward-predictive cues, and thus also the acquisition of secondary rewards, is regulated by prediction error coding. Underlying neural correlates have been consistently demonstrated within the nucleus accumbens (Nacc) as a core region of the mesolimbic dopaminergic system (4, 5). Specifically, receipts of unpredicted rewards are related to increased neural activations and phasic dopaminergic responses within the Nacc and led to behavioral learning with the occurrence of reward (4). On the contrary, the omission of predicted rewards is accompanied by an attenuated neural signaling (6) and extinction of the corresponding behavior with the loss of incentive value. Whereas the pivotal role of the neuromodulator dopamine (DA) has been extensively investigated regarding reinforcement learning and prediction error processing (5, 7, 8), evidence regarding the modulation of the Nacc-activations by other neurotransmitters and under presence of various reinforces is scarce.

We previously investigated the neural correlates of primary reinforcers by functional magnetic imaging (fMRI) and visual erotic video clip stimulation under the selective serotonin-reuptake inhibitor (SSRI) paroxetine and the selective dopamine and noradrenaline reuptake inhibitor (SDNRI) bupropion compared to placebo in healthy subjects (9). Whereas, subjective sexual functioning and corresponding neural substrates were unimpaired or even increased under bupropion, attenuated neural activations were found within neural networks associated to motivational and emotional aspects of sexual behavior accompanied by decreased subjective sexual functions under the SSRI. Specifically, neural activations within the Nacc were diminished under the serotonergic agent (9), potentially due to increased reciprocal interactions with the orbitofrontal cortex (10). Investigations in the same sample of healthy subjects under these two agents, bupropion and paroxetine (11), however, revealed that the serotonergic dampening of the neural reward system is potentially restricted to primary reinforcers, i.e., rewards with an intrinsic, not learned reward value such as visual erotic stimuli (12), and was not evident in a monetary incentive task. For the secondary rewards, signals related to prediction error processing were even more pronounced. Thus, we suggested that neural reward system activations within the Nacc may differentially be modulated by serotonergic agents depending on the context.

To complement our findings on serotonergic and dopaminergic/noradrenergic agents, we further investigated neural correlates of primary rewards by fMRI and visual erotic stimulation under the antipsychotic amisulpride and the selective noradrenaline reuptake inhibitor (SNRI) reboxetine compared to placebo in healthy subjects (13, 14). Here, subjective sexual behavior along with corresponding neural activations were not altered by amisulpride compared to placebo, presumably due to its prodopaminergic properties under low dosages (200 mg/d) (15, 16) as used in this study. Indeed, neural activations within regions corresponding to motivational and emotional aspects of sexual behavior were attenuated under the noradrenergic agent reboxetine accompanied by decreased subjective sexual functioning (14). However, a general dampening of the neural reward system e.g., within the Nacc under reboxetine as observed in this study with primary rewards (14), seemed not intuitive considering the role of the noradrenergic system in the optimization of reward-seeking behavior (17), learning rate (18), and prediction error (19, 20).

Thus, we now examined neural correlates of secondary reward and prediction error processing under reboxetine and amisulpride compared to placebo within the same healthy study sample in which we recently investigated the hedonic aspects of primary rewards by visual erotic picture stimulation (14). Paralleling our previous investigations on serotonergic agents (11), we focused on the Nacc as core region of the mesolimbic dopaminergic system (4, 5) and expected unimpaired neural prediction error signals under the noradrenergic antidepressant reboxetine. The hypothesis was guided by findings of some anatomical overlap of the serotonergic and noradrenergic system (21) and the clinical observation that successful outcomes in environments requiring learning and adaptation of behavior such as behavioral psychotherapy, are even facilitated by antidepressants (22).

## Materials and methods

### Participants

We investigated 20 healthy, heterosexual, male, right-handed subjects under a sub-chronic medication with amisulpride (AMS), reboxetine (REB), and placebo (PLA) in a randomized counterbalanced order as Graf et al. (14). Due to cerebral pathology (gliotic lesions) in one subject and technical MRI-artifacts during the reward paradigm in two subjects, we had to exclude three subjects from further analyses, resulting in a final sample size of 17 participants. The mean age was 23.8 years (SD 3.2; range 20–32 years). Each participant received a full medical evaluation including medical history, physical examination, and a Structured Clinical Interview for DSM-IV Axis I Psychiatric Disorders (SCID-I) prior to the study. Participants with any current or past psychiatric disorder were excluded from the study. Laboratory blood-tests and electrocardiograms were performed to exclude renal, hepatic or cardiac pathology. Further exclusion criteria were any serious general medical condition, any regular medication, current or past neurological illness, relevant baseline sexual dysfunction or sexual disorders, use of illegal drugs and excessive consumption of caffeine or alcohol (>14 units/week). Three (17.64%) of the 17 participants were occasional or moderate smokers. In detail, one subject smoked 5 cigarettes a day, whereas the two remaining subjects smoked 10 or 15 cigarettes a day, respectively. The Massachusetts General Hospital Sexual Functioning Questionnaire [MGH-SFQ; (23)] was administered to evaluate baseline sexual interest, sexual arousal, the ability to achieve orgasm, ability to achieve and maintain an erection, and overall sexual satisfaction prior to the study. According to the study protocol, the questionnaire was modified to assess changes in subjective sexual functioning only over the past week of medication (9, 13). The study was approved by the local ethical committee of Ulm University and all participants gave written informed consent according to the Declaration of Helsinki.

### Study design and procedures

Within a randomized, double-blind, placebo-controlled within-subject crossover design, subjects received 200 mg AMS (100 mg twice per day), 4 mg REB (2 mg twice per day), and PLA (twice per day) for 7 days each. To avoid potential confounds related to the order of drug intake, the study medication was administered in a randomized, counterbalanced order. Intake periods were separated by a wash-out time of at least 2 weeks corresponding to ~21.8 half-life's of REB (24) and 28 half-life's of AMS (25). Subjects were investigated on three different occasions. fMRI-scans took place on the seventh day of medication, 2 h after intake of the last capsule. Subjects were asked to refrain from alcohol parallel to the study medication and to refrain from coffee and nicotine on the day of the scans. To warrant drug exposure and adherence, blood samples were obtained after each scan (about 3 h after drug intake) and analyzed after completion of the whole study. The average plasma AMS-level of the now investigated 17 subjects was 137.2 ng/ml (SD 58.0), the mean plasma REB-level was 75.1 ng/ml (SD 30.3). Blood levels within the expected range were detected in each of the 17 subjects for either drug, indicating that adherence to drug intake was consistent across subjects.

### Additional questionnaires

Sexual functioning during the past week of drug administration was assessed by the MGH-SFQ after each scan. The MGH-SFQ consists of 5 questions with ratings from 1 to 6. Cumulative ratings range from 5 (minimal value: improvement of sexual functioning) over 10 (sexual functioning unchanged compared with normal) to 30 (maximal value: sexual functioning markedly impaired compared with normal). Ratings of more than 2 for single questions, or a cumulative score above 10 indicated subjectively impaired sexual functioning [for details see (9, 23)]. Sedative effects of the medication were assessed with the Stanford Sleepiness Scale [SSS; (26)]. An analyse of variance (ANOVA) for repeated measures and *post-hoc* Newman Keuls tests were computed to analyse questionnaire results.

### fMRI stimuli

**Figure 1:**
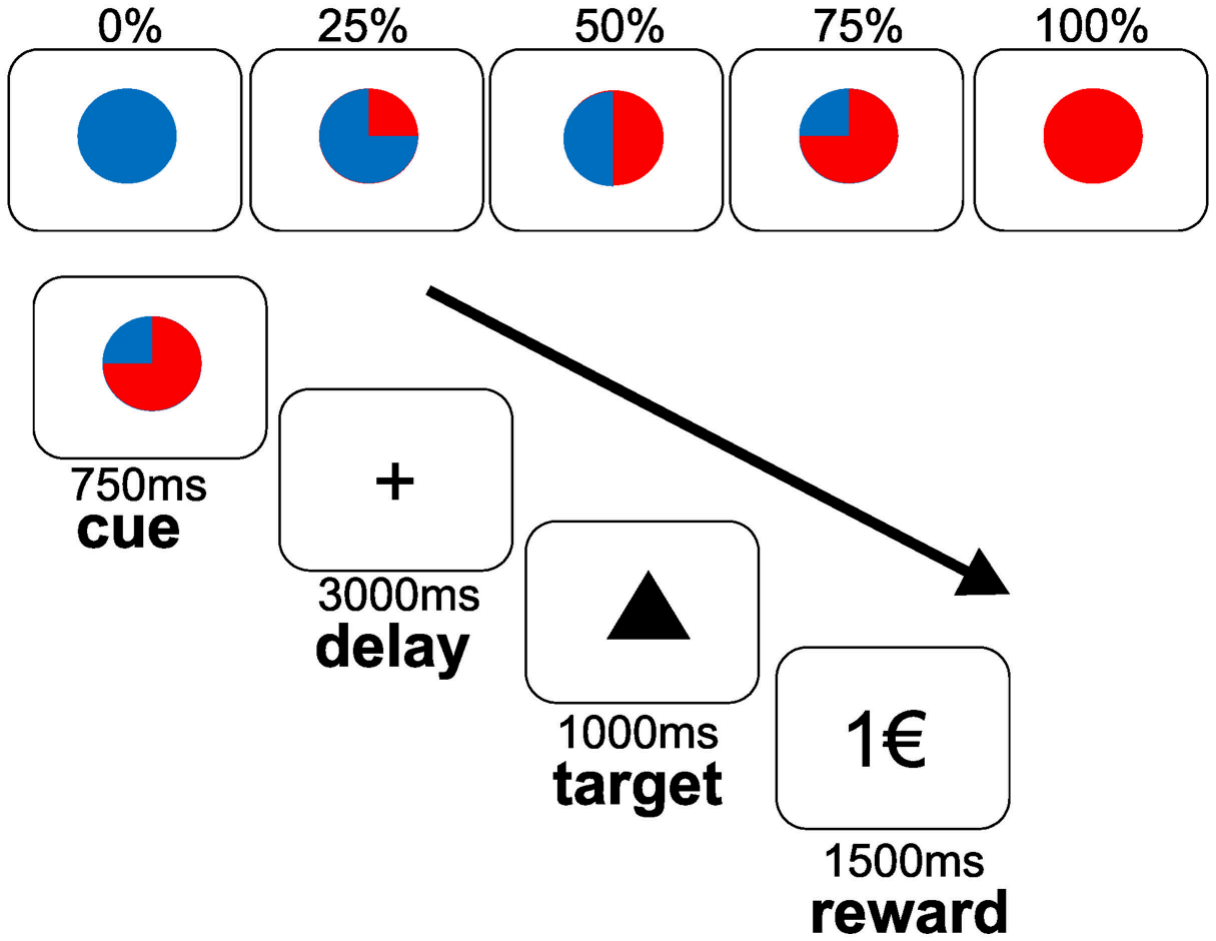
Monetary incentive reward paradigm during fMRI. The upper panel depict cues that indicated the varying reward probabilities (0, 25, 50, 75, and 100%). The lower panel depicts the course of the task. Subjects were instructed to expect a reward of 1€ at the announced probability (cue) during the delay (expectancy period). After the presentation of a target stimulus (triangle as shown or a square), participants had to response by a button press on a two-button box with their right index finger to the triangle target or with their right middle finger to the square target within a fixed time threshold of 1,000 ms. Reward was displayed for 1,500 ms with regard to the previously announced probability (outcome period).

We used an established monetary reward paradigm (6) with a parametric variation of probabilities (0, 25, 50, 75, and 100%) to win 1 Euro (€) (see Figure 1). Taking into account that this investigation was conducted within a broader study design (13, 14), the monetary reward paradigm had to be shortened to reduce total scanner time. In contrast to 120 trials applied in previous studies conducted with the same task (6, 27, 28), each subject completed 60 trials (6,250 ms each), 12 per each probability in a randomized order. The trials started with a cue (colored symbol) that indicated the probability to win the money later on. After an expectation period (delay, 3,000 ms), subjects had to correctly press a button with their index or middle finger for each of two different target symbols (triangle or square) on a two-button box (square: right button; triangle: left button). Correct responses within a fixed reaction time window of 1,000 ms preserved the previously announced chance to win 1€. Feedback (outcome) followed the targets disappearance and notified subjects about the amount of money (1€ or 0€) they won. Reaction times of correct responses within the time window, errors (false button press within the time threshold) and misses (responses out of time window) were registered. According to the reward probabilities, subjects were not rewarded despite pressing the correct button in a number of trials, i.e., a reward announced at a probability of 75% was distributed in 75% (receipt) and held back in 25% (omission) of the correct trials. Incorrect button presses (errors) resulted in a feedback of 0€ at any probability. To ensure a response in all trials, subjects were informed that they would lose 1€ if no button press occurred. Mean reaction times of correct responses within the time window were analyzed by ANOVAs for repeated measures with the main factors “level of probability” (25, 50, 75, 100%) and “treatment” (PLA, AMS, REB). Further, we analyzed numbers of errors and misses between treatment conditions and irrespective of reward probability levels using a similar ANOVA for repeated measures.

For visual erotic stimulation, we used an established erotic picture paradigm as reported in detail in our previous study (14). Briefly, stimuli comprised of 20 erotic and 20 non-erotic pictures of positive emotional content taken from the International Affective Picture System [IAPS, (29)]. Erotic pictures depicted heterosexual couples in erotic poses. Non-erotic pictures showed people engaged in emotionally laden, but non-erotic activities. Pictures were matched for sexual intensity (30) and standard values of arousal, pleasantness and dominance as provided from the IAPS. Erotic and non-erotic stimuli were presented for 4 s (picture perception period) each, followed by a variable inter-stimulus interval with presentation of a fixation cross for 7.5 up to 10.5 s. Half of the stimuli of each condition (erotic and non-erotic) were announced by the presentation of an arrow that allowed the additional investigation of preceding attention prior to sexual stimuli. However, the investigation of preceding attention was not the topic of the current study. Corresponding results are reported in Graf et al. (14).

### fMRI acquisition

Anatomical T1 and functional images were acquired on a 3 Tesla Magnetom ALLEGRA Scanner (Siemens, Erlangen, Germany). During both tasks (the erotic picture and the monetary incentive paradigm), 23 transversal slices were acquired with an image size of 64 × 64 pixels and a field of view of 192 mm. Slice thickness was 3 mm with a 0.75 mm gap resulting in a voxel size of 3 × 3 × 3.75 mm. Images were centered on regions of interest including basal ganglia and prefrontal regions. Five hundred and fifty-two volumes were obtained during the presentation of the erotic picture paradigm and 401 volumes during the monetary incentive task at a TR of 1,500 ms (TE 35 ms, flip angle 90°).

### fMRI analysis

fMRI-data obtained during the erotic picture paradigm were reanalyzed with respect to the different sample sizes (19 vs. 17) to warrant comparability with investigations conducted with the monetary reward paradigm. Image processing and statistical analyses were carried out using Statistical Parametric Mapping (SPM12, Wellcome Trust Centre for Neuroimaging, London, UK) with a random effects model for group analyses. Preprocessing of individual functional images included realignment, slice timing, normalization to a standard template (Montreal Neurological Institute, MNI), smoothing (8 mm FWHM Gaussian kernel) and high pass filtering. After preprocessing, first level analyses were performed for each subject.

According to the general linear model, we defined regressors for each of the two types of pictures presented (erotic, non-erotic) irrespective of their announcement. Picture trials were modeled as timely extended events and convolved with the hemodynamic response function (HRF). The six realignment parameters modeling residual motion were added to the design matrix. For the monetary reward paradigm, we defined regressors for each of the five types of expectation periods sorted by reward probabilities of 0–100%, the button press and the eight different types of outcomes as Abler et al. (6). Depending on the preceding reward expectation (0-100%) and actual outcome (receipt of reward: R; omission of reward: O) the eight outcome events were: 0%, 25%R, 25%O, 50%R, 50%O, 75%R, 75%O, and 100%. Also here, trials were modeled as timely extended events according to their actual durations and convolved with the HRF. The six realignment parameters modeling residual motion were also included in the individual models. On the first level, conditions were weighted with a linear contrast to model neural activations related to increasing reward expectation with increasing probabilities and a linear contrast to model activation following prediction error theory. Based on a linear relationship between brain activity and prediction error as coded by reward probability (6), activations within the Nacc are modeled highest with the most positive prediction error. Thus, when subjects expect to win at a probability of only 25% and win. Nacc activations are modeled lowest with the most negative prediction error, i.e., when subjects expect to win at a probability of 75%, but do not win. In between, Nacc activations decrease linearly. At 0 and 100% probabilities, predictions are definite and no errors occur.

According to our hypothesis and considering the reduced number of trials in the monetary reward paradigm (see above), we focused on a region in interest (ROI) analysis of the nucleus accumbens (Nacc) and defined two ROIs for the left and right Nacc using the masks provided by the Harvard-Oxford cortical and subcortical structural atlases. The left-sided Nacc-ROI comprised of 74 voxels (voxel-size: 2 × 2 × 2 mm), the right-sided ROI consisted of 63 voxels. The binary right- and left-sided Nacc masks were then combined by using imcalc as provided by SPM.

Similar to our previous investigation in the same study sample (14), a two-factorial analysis of variance (ANOVA) for repeated measures was computed with the factors condition (erotic, non-erotic) and treatment (PLA, AMS, REB) within the inclusive Nacc-mask at a statistical threshold of *p* < 0.05 FWE-corrected, for size of search volume. Regarding the reward paradigm, individual weighted contrast images were analyzed by an ANOVA for repeated measures (*F*-test on treatment effects) within a full factorial design as implemented in SPM and within the inclusive Nacc mask at a statistical threshold of *p* < 0.05 (FWE-corrected) for the expectancy and the outcome period.

According to our hypothesis regarding an unimpaired Nacc activation during the monetary reward task in contrast to the erotic picture paradigm under REB, we computed one-sample *t*-tests on parameter estimates of the left and right Nacc. Notably, these *t*-tests were not conducted to perform *post-hoc* comparisons between treatments but to demonstrate significantly positive effects of task (reward expectation and prediction error processing) under REB.

In addition to the ROI analyses and for exploratory purposes, a whole brain analysis was conducted on effects of task irrespective of treatment condition. Please note that the methods and results regarding this whole-brain analyses are detailed in our Supplementary Material.

## Results

### Questionnaires

An ANOVA for repeated measurements revealed no significant treatment effects on sedation or sleepiness assessed by the SSS immediately after fMRI scanning [*F*_(2,32)_ = 0.84; *p* = 0.442]. The mean overall score in the MHG-SFQ upon enrolment was 10.8 (SD 1.79) and 11.6 (SD 2.48) under PLA. Paired *t*-testing revealed no significant difference between PLA and enrolment scores [*t*_(1, 16)_ = −1.22; *p* = 0.241], thus MGH-SFQ-data upon enrolment were not considered further. Treatment effects on MHG-SFQ sum-scores were significant [*F*_(2,32)_ = 7.76; *p* = 0.002] and *post-hoc* Newman Keuls confirmed more impaired sexual functioning under the REB compared to both, PLA (*p* = 0.004) and AMS (*p* = 0.003). Sexual functioning under AMS did not differ from PLA (*p* = 0.635). Considering the different subscales, we found significant treatment effects on all subscales (*p* < 0.05). Here, *post-hoc* Newman-Keuls revealed detrimental effects of REB relative to AMS regarding sexual satisfaction whereas attenuated sexual interest, sexual arousal, ability to achieve orgasm and to achieve/maintain an erection was observed under REB compared to both, PLA, and AMS. As already observed in our previous investigation in 19 subjects (13, 14), comparisons between AMS and PLA did not reveal significant differences in any of the subscales.

### Behavioral responding during the reward paradigm

**Figure 2:**
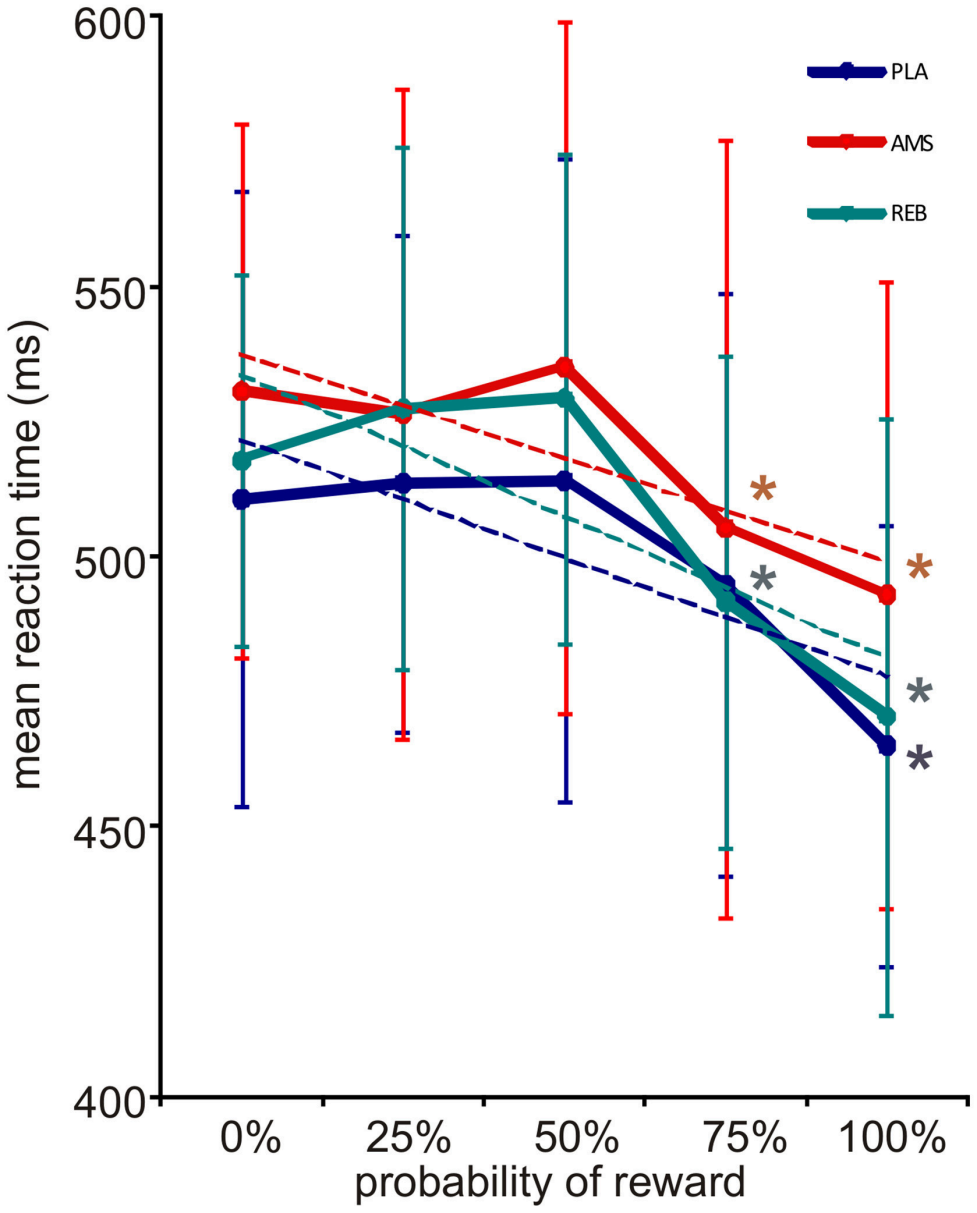
Reward expectation and corresponding reaction times (with standard deviations). Mean reaction times of the fMRI reward paradigm accelerated under all treatment conditions according to increased reward probability (blue = placebo, PLA; red = amisulpride, AMS; green = reboxetine, REB). Dotted lines depict linear trendlines for each treatment condition. An ANOVA revealed significant results for the factor level of probability (see Results section). In *post-hoc* testing, a significant acceleration of reaction times was observed when comparing 0 to 100% under PLA and 0 to 75% reward probability under AMS and REB. *Indicate statistical significance (*p* < 0.05) in *post-hoc t*-testing.

During the reward paradigm, subjects responded correctly and within the requested time in 97.8% of trials under PLA and in 98.8% of trials under both, AMS and REB. An ANOVA on correct trials did not reveal significant treatment effects [*F*_(2, 32)_ = 1.77; *p* = 0.186]. Further, misses [*F*_(2, 32)_ = 0.46; *p* = 0.636] or errors [*F*_(2, 32)_ = 1.48; *p* = 0.242] in trials did not significantly differ between treatments. An ANOVA for repeated measures on mean reaction times of correct responses revealed a significant main effect for levels of probability [*F*_(4, 64)_ = 27.26; *p* = 0.000], whereas no significant results could be observed for the main factor treatment [*F*_(2, 32)_ = 1.20; *p* = 0.314] nor the interaction of the main factors [*F*_(8, 128)_ = 0.67; *p* = 0.719]. Mean reaction times of correct responses decreased according to higher reward probability in all treatment conditions (see Figure 2). Here, *post-hoc t*-testing on these reaction times according to different levels of reward probability within each treatment condition revealed a significant mean reaction time acceleration under PLA when comparing 0–100% reward probability (PLA: 0–25%: *p* = 0.725; 0–50%: *p* = 0.747; 0–75%: *p* = 0.062; 0–100%: *p* = 0.000). Under AMS and REB, mean reaction times accelerated significantly from 0 to 75% (AMS: 0–25%: *p* = 0.571; 0–50%: *p* = 0.604; 0–75%: *p* = 0.020; 0–100%: *p* = 0.001; REB: 0–25%: *p* = 0.342; 0–50%: *p* = 0.149; 0–75%: *p* = 0.037; 0–100%: *p* = 0.002). Mean reaction times of correct responses in conditions with 0 and 100% reward probability, respectively, did not differ between PLA, AMS, and REB (all *p* > 0.05).

### fMRI results

Although we now included a smaller sample size of 17 participants in contrast to our previous analyses in 19 subjects (14), we could confirm our results regarding neural activations during erotic static picture stimulation by significant (*p* < 0.05 FWE-corrected) treatment-by-condition interaction effects revealed from the ANOVA within the bilateral Nacc. Here, *post-hoc* comparisons (*F*-tests) demonstrated a significant (*p* < 0.05 FWE-corrected) attenuated neural activation within the bilateral Nacc under REB compared to PLA. No significant differences in neural activations under static erotic picture stimulation were observed comparing AMS with PLA or REB.

**Figure 3:**
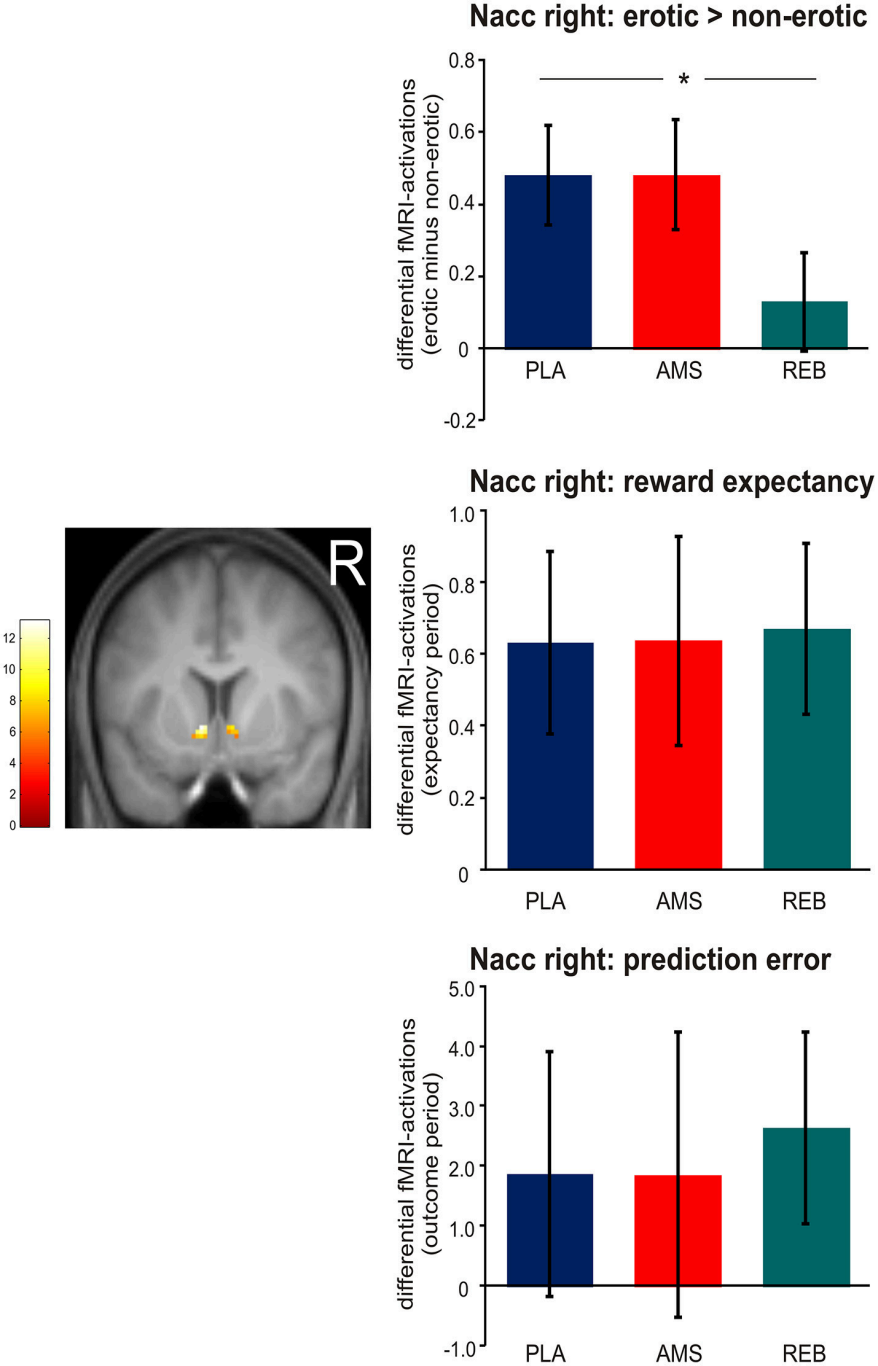
Neural activations within the right nucleus accumbens (Nacc) observed during static visual erotic picture stimulation and during the monetary reward paradigm. For demonstrational purposes, parameter estimates were extracted solely from the right Nacc. The brain image depicts differential (erotic minus non-erotic) neural activations within the Nacc mask provided by the Harvard-Oxford cortical and subcortical structural atlas during the static erotic picture paradigm. Bar graphs depict differential neural activations with standard error of the mean (sem). Corresponding parameter estimates were extracted only from the right Nacc cluster for demonstrational purposes. In detail, the upper panel shows differential (erotic minus non-erotic) neural activations under placebo (PLA), amisulpride (AMS), and reboxetine (REB). Here, *post-hoc t-*tests revealed significant attenuated fMRI-activations under REB vs. PLA (*statistical significance). The middle panel depicts differential fMRI-activations (individually weighted by linear contrasts, see Materials and Methods section) during the expectancy period in the monetary reward paradigm. Bar graphs in the lower panel show differential fMRI-activations modeling prediction error (also weighted with linear contrast).

Regarding the reward paradigm, an ANOVA for repeated measures revealed no significant treatment-by-condition interaction effects (*p* < 0.05 FWE-corrected) on neural activation during both, expectancy or outcome periods (see Figure 3).

Although the lack of treatment effects during the monetary reward task in contrast to the erotic picture paradigm already supported our hypothesis, one sample *t*-tests were computed to examine whether differential effects of reward expectation and prediction error processing under REB were significant (positive effect of task). We observed no significant positive effect of task in the right Nacc during erotic picture stimulation (*p* = 0.178) under REB. In contrast, we found a significant positive effect of task in the right Nacc under monetary reward expectancy (*p* = 0.006) and showed a trend to significance under prediction error processing (*p* = 0.060). Further, significant differences in neural activations were found within the left Nacc during reward outcome (*p* = 0.033).

## Discussion

In a slightly smaller sample of subjects, we could confirm our previous results (14) of attenuated neural activations within the Nacc under the noradrenergic agent REB along with diminished subjective sexual functioning compared to PLA. Further, AMS left neural activations within the Nacc along with subjective sexual behavior unimpaired. Analyses of the monetary incentive paradigm revealed an acceleration of reaction times of correct responses upon increasing reward expectation in all treatment conditions. Accordingly, neural activations modeling prediction error learning signals as well as monetary reward expectancy within the Nacc were unimpaired under both, AMS and REB, in contrast to our investigations conducted with erotic picture stimuli as primary reinforcers.

An acceleration in reaction times according to increased monetary reward expectation has been observed in previous investigations in healthy subjects conducted with the same fMRI-paradigm (6). However, treatment effects on reaction times were not evident, neither for REB nor AMS compared to PLA. This is in line with investigations demonstrating unimpaired reaction times under AMS in a reward-based choice reaction task even under higher dosages as used in our study (31). Further, our results regarding comparable reaction times under REB and PLA in our monetary reward paradigm are in accordance with unimpaired reaction times under REB in a choice reaction task (32) and in a diminishing-utility task with increases in reward value (33). Considering the attenuated subjective sexual functioning under REB in the light of otherwise unimpaired behavioral results during monetary reward as found in our study, these results may support that noradrenergic agents rather attenuate behavioral responses to primary rewards while behavioral responding to secondary reinforcers are left unimpaired. Alternatively, noradrenergic agents may solely alter consummatory aspects of reward whereas reinforced learning remains unchanged.

The idea of a differential noradrenergic modulation of the human reward system regarding primary vs. secondary reinforcers is not only supported by these behavioral results, but also by diverging neural alterations in the presence of erotic vs. monetary stimuli. Regarding visual erotic stimulation, we could confirm our previous results (14) of significant treatment-by-condition interaction effects within the bilateral Nacc and attenuated neural Nacc activations under REB compared to PLA in *post-hoc* testing, now using an ROI approach in a slightly smaller sample. In contrast, significant treatment effects on neural Nacc activations were not evident in the monetary incentive task. Effects of reward expectation and prediction error processing were significant under reboxetine. Hence, we could confirm our hypothesis of unimpaired neural secondary reward and prediction error signaling under the noradrenergic antidepressant REB. This finding is of great relevance considering the attenuated neural reward-related prediction error signal in untreated depression (34) where further impairments would be undesirable. The same holds regarding the fundamental role of prediction error processing in behavioral learning and decision-making (35), both essential features in psychotherapy.

Our findings of unaffected neural prediction error signals under noradrenergic agents are in line with a previous study investigating learning rates modeled as prediction error processing in healthy subjects under the chemically similar noradrenergic agent atomoxetine (20). Beneficial effects on learning rates were observed under atomoxetine following unanticipated task changes as also implemented in our monetary reward paradigm, supporting the pivotal role of the noradrenergic system in the adjustment or reinforced learning following environmental changes (17). However, our interpretations regarding the neural correlates of primary rewards under REB compared to PLA in healthy subjects are contradictory to the findings of one study that revealed unaffected Nacc activations upon the processing of rewarding food stimuli under this agent (36). Though, in this study, attenuated neural activations under REB were also found within the medial orbitofrontal cortex, previously related to the coding of pleasantness or received reward (37). Evidence supporting the interpretation of a differential modulation of primary erotic and secondary monetary rewards within the Nacc (11) highly similar to the current study was also found in our previous study with the SSRI paroxetine. The similarity of the findings under the noradrenergic agent REB as found in this study may be plausible considering some anatomical overlap of these two neuromodulatory pathways (21). However, the precise underlying mechanisms are still subject for further research.

With unimpaired subjective sexual functioning and unchanged neural activations within the Nacc during visual erotic stimulation under AMS compared to PLA, we further confirmed our results even in a smaller sample size (14). The absence of treatment effects on neural correlates of erotic stimulation but also secondary rewards as in the current study may relate to our previous interpretation of prodopaminergic effects of AMS in lower dosages (15, 16) as used in our study. Another study also observed no significant treatment effects on reinforcement learning under the same dosage of AMS (200 mg), but enhanced striatal prediction error coding in a value-based choice task (31). In contrast, attenuated appetitive prediction error signaling was demonstrated at higher dosages of AMS (400 mg) (38) and support our interpretation. The observation that agents with pure antidopaminergic properties such as haloperidol decreased Nacc activations during a sexual stimulation task (39) but also reinforcement learning and striatal prediction error coding (35), suggest that higher dosages of AMS might dampen the human reward system irrespective of the presence of primary or secondary reinforcers. However, this conclusion remains speculative and should be a subject of further research.

### Limitations

Considering that our investigations on neural alterations of the noradrenergic antidepressant REB and the antipsychotic drug AMS took place in healthy subjects, transferability of our results to patient populations may be limited. Otherwise, investigating a sample of healthy subjects allowed us to assess mere drug effects without confounds arising from the disorder itself. However, generalizability to the population may be limited by our small sample size.

Taking into account that this investigation was conducted within a broader study design (13, 14), the monetary reward paradigm consisted of a total of 60 instead of 120 trials previously applied (6), to reduce total scanner time. Thus, the reduced number of trials may have generated weaker BOLD-signals resulting in a smaller power to detect treatment effects. Although only 3 of the 17 participants were occasional or moderate smokers, and were asked to refrain from nicotine on the day of the MRI-scans, our results might be confounded by the smoking status considering the effects of nicotine on the human reward system (8). However, inclusion of smoking status as a covariate left results unchanged. Considering that we used a parametric statistical approach with clusterwise inference, we cannot fully exclude a higher degree of false positive results (40). Thus, our results await empirical replication.

## Conclusion

We previously demonstrated attenuated neural activations within the nucleus accumbens and diminished subjective sexual functioning under the noradrenergic agent reboxetine compared to placebo. However, a global dampening of the human reward system under antidepressant medication seemed not intuitive from a clinical perspective and motivated our investigations regarding the effects of reboxetine on neural processing of secondary rewards. Given the unimpaired behavioral and corresponding neural responses regarding monetary reward expectation and prediction error signaling, our results suggest a specific and not a global effect of noradrenergic agents such as reboxetine on neural reward processing potentially related to the presence of either primary or secondary reinforcers. Considering the pivotal role of prediction error processing in reinforcement learning, our results support the neutral or even facilitating effects of noradrenergic agents in environments requiring behavioral learning and adaption, e.g., psychotherapeutic treatment.

## Author contributions

HG and BA conception and design, acquisition, analysis, interpretation of data, drafting the work, final approvement, accountable for all aspects of the work. MW acquisition, analysis, interpretation of data, revising the work, final approvement, agreement to be accountable for all aspects of the work. CM design, analysis, interpretation of data, drafting the work, final approvement, accountable for all aspects of the work. MWa conception and design, analysis, interpretation of data, drafting the work, final approvement, accountable for all aspects of the work.

### Conflict of interest statement

The authors declare that the research was conducted in the absence of any commercial or financial relationships that could be construed as a potential conflict of interest.
