# Supplementary material for: Differential Noradrenergic Modulation of Monetary Reward and Visual Erotic Stimulus Processing
Source: Front Psychiatry. 2018 Jul 31;9:346. doi: 10.3389/fpsyt.2018.00346 (PMC6079271; doi:10.3389/fpsyt.2018.00346)
Supplement: Supplementary file 1 [file Data_Sheet_1.docx]

**Supplementary Material**

**Differential noradrenergic modulation of monetary reward and visual erotic stimulus processing**

Heiko Graf¹*, Maike Wiegers¹, Coraline D. Metzger^2,3,4^, Martin Walter^5^_,_ Birgit Abler¹

¹ Department of Psychiatry and Psychotherapy III, Ulm University, Ulm, Germany

² Department of Psychiatry, Otto von Guericke University, Magdeburg, Germany

^3^ Institute of Cognitive Neurology and Dementia Research (IKND), Otto von Guericke University, Magdeburg, Germany

^4^ German Center for Neurodegenerative Diseases (DZNE), DZNE site Magdeburg, Germany

^5^ Department of Psychiatry, Eberhard Karls University, Tuebingen, Germany

**Whole-brain analyses - Methods**

Apart from our ROI-analysis, we computed a whole-brain analysis for exploratory purposes on main effects of task considering the reduced number of trials in our monetary reward paradigm. An analysis on whole brain effects of task was computed at a statistical threshold of p<0.05 (FWE-corrected on cluster level) for the monetary expectancy, and for the outcome period at a lenient threshold of p<0.005 uncorrected. Within these significant cluster, treatment effects were examined by omnibus F-tests for the reward expectancy and the outcome period, respectively. Moreover, we further explored the whole-brain task effects during the PLA condition at a statistical threshold of p<0.05 (FWE-corrected on cluster level) for the monetary expectancy, and for the outcome period at a lenient threshold of p<0.005 uncorrected.

**Whole-brain analyses - Results**

For the monetary expectancy, increased neural activation irrespective of treatment conditions were found within the bilateral inferior frontal gyrus, the anterior and midcingulate cortex, the Nacc, the ventral tegmental area and the midbrain, as well as in the occipital cortex and the cerebellum. An omnibus F-test within this inclusive and binary mask did not reveal any significant treatment effects under the same statistical threshold (p<0.05 FWE-corrected on cluster-level). However, neural activations regarding prediction error processing were not evident when applying this threshold including a correction for multiple comparisons. Presumably due to the reduced numbers of trials, we revealed weaker task effects by increased neural activations within the right orbitofrontal cortex and right midbrain irrespective of treatment under the mentioned statistical threshold of p<0.005 uncorrected. Also here, an omnibus F-test within this inclusive and binary mask did not reveal any significant treatment effects.

Moreover, we examined whole-brain effects of task solely under placebo and revealed an increase of activity (p<0.05 FWE-corrected on cluster-level) within the left inferior frontal gyrus, the left temporo-parietal junction, the anterior cingulate cortex and the left occipital cortex during monetary reward expectancy. Similar to our analysis of condition effects irrespective of treatment, we found increased activations within the right orbitofrontal cortex, the midbrain, the anterior cingulate cortex, the right temporal gyrus, the occipital cortex and the cerebellum only at a lenient threshold of p<0.005 uncorrected.
